# Digitalis

**Published:** 1842-02

**Authors:** 


### DIGITALIS.

Dr. Calvin Jones, of West Tennessee, a physician of age and experience, has sent us a paper designed to recal the attention of his brethren to the value of Digitalis, as a remedy in pulmonary and dropsical diseases. We at first thought of publishing it entire; but have concluded, without intending the least disrespect to its worthy author, to give it in abstract.

He begins by expressing the opinion that Digitalis is a "remedial agent of great power, for which a substitute could not easily be obtained;" and then proceeds to inquire into the causes which, in latter times, have limited its use. They are, first, its being frequently inert, and of course doing no good; second, its having been used under inappropriate states of the system, when it has done harm. Of these Dr. Jones justly regards a high phlogistic diathesis as the most common. He cites a case of phthisis pulmonalis, in which this medicine appeared to prolong life for many years; but as it occurred before auscultation was invented, or pathological anatomy formed into a science, we have no means of knowing whether it was tubercular consumption, or only chronic bronchitis. In the latter, digitalis is certainly one of our best remedies. In the treatment of dropsies, Dr. J. has found this medicine of great value,—provided venesection preceded it. The following case, on account of its remote cause, we extract in the Doctor's own words:—

"I will mention but one other case, though several present themselves. Alexander Hobby, of Johnston County, N. Carolina, (I then resided on the Neuse,) for three or four years, in the cider and apple-brandy seasons, after considerable use and abuse of those articlesh ad attacks of general dropsy. I uniformly bled him largely, gave him liberal quantities of nitrous salts, and, when his pulse became soft and yielding, completed the cure with digitalis. I mention as evidence of the usual salutary effect of the medicine, and the power which the habit of observation gave of predicting it, that I once told him that on such a morning I should visit him, when his pulse would be reduced from its 90 to 42 strokes in a minute. When he found the prediction exactly verified, (the precise number was a random guess of course), he was alarmed with the supposition that he was surrounded with supernatural intelligences and influences; a common belief among certain people in those bye-gone days, (1799) when the author of evil was strongly suspected of doing great good to the sick by means of his purchased agents. A cider season at last came, when I was necessarily absent from home, (in attendance in the Legislature of which I was a member), and poor old Hobby became its victim."

Of the preparations of Digitalis Dr. J. prefers the saturated tincture of the recent herb—from 12 to 16 drops twice a day, to be laid aside when the characteristic constitutional effects appear, and resumed when they have passed away.

